# Pelvic incidence variation among individuals: functional influence versus genetic determinism

**DOI:** 10.1186/s13018-018-0762-9

**Published:** 2018-03-20

**Authors:** Hong-Fang Chen, Chang-Qing Zhao

**Affiliations:** Shanghai Key Laboratory of Orthopaedic Implants, Department of Orthopaedics, Shanghai Ninth People’s Hospital, Shanghai Jiao Tong University School of Medicine, 639 Zhizaoju Rd, Shanghai, 200011 People’s Republic of China

**Keywords:** Pelvic incidence, Spinal surgery, Bipedal locomotion, Genetics

## Abstract

Pelvic incidence has become one of the most important sagittal parameters in spinal surgery. Despite its great importance, pelvic incidence can vary from 33° to 85° in the normal population. The reasons for this great variability in pelvic incidence remain unexplored. The objective of this article is to present some possible interpretations for the great variability in pelvic incidence under both normal and pathological conditions and to further understand the determinants of pelvic incidence from the perspective of the functional requirements for bipedalism and genetic backgrounds via a literature review. We postulate that both pelvic incidence and pelvic morphology may be genetically predetermined, and a great variability in pelvic incidence may already exist even before birth. This great variability may also serve as a further reminder that the sagittal profile, bipedal locomotion mode, and genetic background of every individual are unique and specific, and clinicians should avoid making universally applying broad generalizations of pelvic incidence. Although PI is an important parameter and there are many theories behind its variability, we still do not have clear mechanistic answers.

## Background

**Fig. 1:**
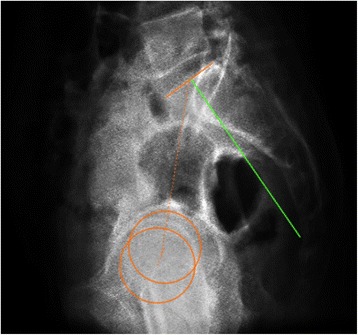
The schematic shows the measurement of pelvic incidence

Pelvic incidence (PI), first introduced by Legaye et al. [1] in 1992, is defined as the angle between the line perpendicular to the sacral endplate at its midpoint and a line connecting this point to the axis of the femoral head (Fig. 1). PI increases gradually during childhood as a result of bipedal walking [2]. After bone maturity, PI remains constant for every individual since the motion of the sacroiliac joint can be ignored [3].

PI is one of the most important anatomic parameters of the pelvis in terms of spinal sagittal balance and equals the sum of the positional parameters sacral slope (SS) and pelvic tilt (PT) (PI = SS + PT) [4]. PI strongly correlates with lumbar lordosis (LL) through SS, thus largely determining the different lumbar types and consequent mechanisms of lumbar spine degeneration [5]. Moreover, the compensatory capacity of PT determined by PI is partly involved in acetabular impingement and hip osteoarthritis [6]. A postoperative mismatch between PI and LL may lead to proximal junctional failure (PJF) after long-segment spinal fusion or adjacent segmental disease (ASD) after short-segment spinal fusion [7]. In addition, decreased PI is reported to increase the risk of prosthetic impingement after total hip arthroplasty (THA) [8].

Despite its importance, there are still unanswered questions about PI. First, why does PI value vary so much among adults, from 33° to 85°? [9] Second, do the functional requirements of bipedalism or the genetically determined developmental potential primarily contribute to the gradual increase of PI during children’s growth? [10] Answering these questions can be helpful for further understanding the relationships among PI, LL, and acetabular orientation for the design of better surgical plans before spinal fusion or hip replacement.

Although PI is an important parameter and there are many theories behind its variability, we still do not have clear mechanistic answers. The object of this review is to present some possible interpretations for the great variability in PI under both normal and pathological conditions. This article may also provide greater insight into the relationship between pelvic parameters and pelvic morphology and further explain the determinants of PI from the perspective of the functional requirements for bipedalism vs. genetic background.

## Functional influence

Increased PI is observed not only in the evolution of bipedal locomotion during human evolution but also in walking acquisition during children’s growth [2, 11]. Human ambulation changes the biomechanical environment in the lumbosacral region; thus, different upright postures or locomotion modes may impact the PI value differently. The following section discusses possible correlations between the formation and increase of PI and the changes in the biomechanical environment around the lumbosacral region due to the functional requirements of normal or abnormal bipedal locomotion.

**Fig. 2:**
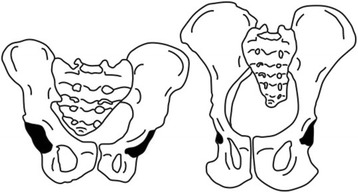
The schematic shows the characteristic of chimpanzee pelvis (right) compared to human pelvis (left)

During the transition from the quadrupedal locomotion of apes to the occasional and finally permanent bipedalism of the hominoids, an increase in PI is observed [12]. Upright posture has led to major biomechanical changes of the human trunk, especially in the lumbopelvic region, including strengthening of the lumbar extensors and changes in the origins and terminations of muscles (including the psoas major, quadriceps femoris, multifidus) [13], since the pelvis connects the upper trunk and lower limbs. Several authors believes that increased PI can be ascribed to the different anatomical structures of the human pelvis adapt to the acquisition of upright walking because there are significant differences in pelvic morphology between humans and other species [14]. Even the pelvis of a great ape, the closest living relative of humans, is remarkably different from the human pelvis. The human pelvis is short in the vertical plane but has a great girth in the horizontal plane, in contrast to a great ape, where the pelvis is elongated vertically but narrow horizontally. The iliac blades in the human pelvis are short and thickened, positioned laterally over hip joint, while the great ape has long, flat iliac blades [15]. The location of the human sacrum is low and behind the acetabulum within the pelvic cavity, and shape of the sacrum is considerably curved. On the contrary, the sacrum in the ape is vertical and directly above the hip joint, and its location within the pelvic cavity is high (Fig. 2). During this evolutionary process, the PI value increases, suggesting that the bipedal mode of locomotion may have had a great impact on pelvic morphology [16]. Nevertheless, the establishment of bipedalism is rather complicated, and the increased number of lumbar vertebrae, the formation of femoral anteversion, and the neck shaft angle are also of great importance in this evolution besides the increase of PI. Moreover, it cannot be ignored that there is a small group of people with a PI that is even smaller than that of the *Australopithecus*, reminding us that the impact of bipedal locomotion on PI may be overestimated [17].

Augmentation of PI is also found during walking development in adolescents before bone maturity. It is generally accepted that there is a positive correlation between age and PI, and PI becomes stable and remains unchanged around the age of 10 [2]. The sacrum in newborns is flat and close to the iliac crest. During growth and walking acquisition, the sacrum becomes curved and far from the iliac crest [18]. Several hypotheses have been proposed to explain the increase of PI and pelvic morphological changes during the acquisition of ambulation in adolescents. The strengthening of the musculature seems to provide an appropriate biomechanical environment around the lumbosacral junction during growth. Two possible mechanisms are involved in the development of the upright position as well as the increase of PI: (1) coxofemoral extension and verticalization of the pelvis both arise from the action of the gluteal muscles, which brings the superior endplate to a more horizontal position, more suitable for weightbearing; (2) horizontalization of the sacrum, effectuated by the erector spinae muscles, bringing the upper sacral endplate into a more vertical position [19]. Interestingly, Hayama et al. [20] observed a significant increase of PI as well as LL in Japanese macaques that were trained for bipedal walking during infancy. PI in these primates is remarkably higher than PI in a quadrupedal macaque. This study may also indicate that erect posture during growth has an impact on PI. Unfortunately, this study did not evaluate the muscle power around the lumbopelvic region of these macaques. Since the process of walking acquisition and an increase in age proceed simultaneously in a growing child, it is difficult to determine which factor accounts for the increasing PI, whether it is the erect posture, the strengthening of the muscles, or the development and maturity of the skeleton. If we recruited a study group of children who had been bedridden ever since birth and never had adopted an erect posture and ambulation, and observed the changes of PI and pelvic morphology during growth, it would be extremely helpful in understanding the formation and increase of PI.

Surprisingly, in ambulatory children with cerebral palsy (CP) whose gait and posture are obviously disturbed owing to muscle impairment around the lumbopelvic region, there is no significant difference in the value of PI between patients with CP and normal children, although significant differences for LL and pelvic rotation have been reported [21, 22]. Massaad et al. [23] indicated that PI was significantly increased in children with CP (7 hemiplegia, 20 diplegia, GMFCS levels I:17, II:10) compared to typically developing children (48° ± 7° vs. 43° ± 8°), although this difference was small.

Studies of PI and pelvic morphology in pathological conditions such as cerebral palsy, kyphotic deformity, and spondylolisthesis may have greater implications than similar studies in a normal population. Kyphotic deformity is thought to cause hyperextension stress at the lumbosacral junction. Patients with Scheuermann’s disease (SD) with kyphotic deformity are compensated by lumbar hyperlordosis, pelvic retroversion, and even cervical hyperlordosis to maintain sagittal balance and keep a horizontal visual gaze [24, 25]. Peleg et al. [26] reported a horizontally orientated sacrum in patients with Scheuermann’s kyphosis. A horizontally orientated sacrum implicates a high PI in a normal population. Subjects with a large PI can normally be classified as type 4 with hyperlordosis of the lumbar spine. This author believed that thoracic hyperkyphosis was a compensatory change to this horizontally orientated sacrum and not vice versa. On the contrary, Jiang et al. [27] and Tyrakowski et al. [28] reported a significantly lower PI in adolescent patients with SD compared to adults with normal spinal alignment. The correlation between thoracic hyperkyphosis and low PI in patients with SD needs to be further elucidated.

One possible explanation is that thoracic and thoracolumbar kyphosis prior to skeletal maturation is likely to hinder the increase of PI. Li et al. [25] studied 49 patients who developed kyphotic deformity at a mean age of 7.1 years. They found that the mean value of PI in these patients was significantly smaller compared with those reported in normal subjects. Moreover, the PI of patients with a kyphotic apex located at the lower thoracic spine or thoracolumbar junction was significantly smaller than that in patients with the apex located in the upper or middle thoracic spine. This is in agreement with the fact that PI in patients with Scheuermann’s thoracic kyphosis (STK) is significantly higher than PI in patients with Scheuermann’s thoracolumbar kyphosis (STLK) [27]. Tyrakowski et al. [28] hypothesized that an increased TK or TLK in patients with SD was first compensated by increased LL. When the compensative capacity of LL was at its limit, any further increase in TK or TLK was compensated by pelvic retroversion that increased PT. Increased PT resulted in a posterior shift of the gravity line that was biomechanically disadvantageous [29, 30]. Compensating for the decrease in SS while avoiding increasing PT in a growing child might result in a modification of the pelvic shape that decreases PI. For patients with a deformity in the upper or middle thoracic spine, the compensatory capacity of LL might be sufficient. In patients with deformity of the lower thoracic spine or thoracolumbar spine, compensation of LL is not sufficient, and thus, pelvic retroversion involvement and hindrance of PI are more prominent (Table 1).

**Table 1:** Comparison of pelvic incidence, pelvic tilt, and sacral slope between patients with Scheuermann’s deformity at different levels

	Kyphotic level	PI (°)	PT (°)	SS (°)
Li’s study [25]	T1–T8	40.2 ± 7.5	− 5.6 ± 13.2	45.9 ± 13.4
	Below T8	32.5 ± 11.9	2.9 ± 10.8	29.9 ± 15.5
Jiang’s study [27]	T1–T10	36.5 ± 8.4	− 1.8 ± 7.6	38.4 ± 7.9
	T11–L1	29.1 ± 11.3	1.4 ± 12.8	28.5 ± 10.9

*PI* pelvic incidence, *PT* pelvic tilt, *SS* sacral slope

It is assumed that bipedalism, as well as biomechanical changes following upright posture, influences PI and pelvic morphology, but PI is not simply determined by bipedal locomotion, and to what extent PI is influenced by this locomotion mode is unknown. Although pelves in the CP population who have never adopted a standing posture after birth are poorly studied and need to be investigated further, PI is not merely determined by the bipedal function of human beings, and the spinal sagittal profile also certainly influences PI and pelvic morphology. This reminds us that genetic factors contribute to the value of PI.

## Genetic determinism

Several authors proposed that the PI value was adapted to the formation of lumbar lordosis during growth, not the other way around [31]. Choufani et al. [32] analyzed 45 MRIs of fetuses aged 23–40 weeks of gestation and concluded that the fetal spine is not formed merely with one curvature, but rather, all the curves mathematically showed the presence of lumbosacral lordosis. This fact may indicate that the sacrum is already tilted posteriorly in the fetus, and the variability of PI already exists even before birth.

Studies have shown the hereditability of the sagittal spinal profile. A study that consists of 110 monozygotic and 136 dizygotic twins suggests that heritability estimates ranged between 41% (19–59%) for PI and 59% (42–71%) for LL [33]. Moreover, it is generally accepted that children of parents with scoliosis may inherit a tendency to idiopathic scoliosis [34]. Moke et al. [35] reported a genetic susceptibility to dysplastic developmental spondylolisthesis in two identical twins from two unrelated families. Scheuermann’s disease has some familial aggregation and shows a certain degree of genetic tendency [36]. A high concordance for SD has also been demonstrated in monozygotic twins. Dryden et al. [37] recruited 446 healthy children aged 10–15 years, consisting of 11 monozygotic twin pairs, 28 dizygotic twin pairs, 98 same-sex sibling pairs, and 86 different sex sibling pairs. This study did show that lordosis or a possible S-shape of the spinal profile had a significant familial correlation (the highest correlation was in monozygotic twins, then dizygotic twins, then same-sex siblings, and finally different sex siblings), and there was a significant correlation in the same-sex pairs but not in the different sex pairings. Thus, they suggested that some elements of the normal spinal profile might be associated with genetic proximity and sex.

These works highlight the concept that genetic factors may have a great influence on spinal curvature. The sagittal balance of the trunk is defined as the optimal lordotic positioning of the spinal column above a correctly oriented pelvis [1]. Characteristics of the spinal curvature ought to be reflected by the pelvic morphology, namely, the value of PI. The genetic background must be taken into consideration because it may predetermine the pelvic morphology.

Boulay et al. [38] measured 12 anatomical pelvic specimens; they reported a negative correlation between PI and pelvic thickness (PTH). Jean et al. [18] also observed significantly higher values of PTH in populations with high-grade spondylolysis than in normal controls. Abitbol et al. [39] suggested that with a large PI, the sacrum is much more curved. The sacrum is rather flat when the PI is small. By simultaneously considering these morphological changes in the pelvis, we can postulate that a high PI value is associated with a greater lordotic lumbar curvature, a strong SS, a more concaved sacrum, a lower position of the sacral plate in relation to the iliac crests, and a smaller distance between the hip axis and sacrum. A small PI angle is coupled with a flat lumbar curvature, a small SS value, a less curved sacrum, a higher position of the sacral endplate in relation to the iliac crests, and a larger distance between the midpoint of the sacral endplate and hip axis. This study agrees with hominid pelvic evolution. Moreover, the great variability of PI may indicate diversities of pelvic morphology acquired from the evolution of bipedal locomotion that may be passed on by heredity.

Although PI formation is multifactorial, genetic factors may play an important role in determining the PI value. The genetic contribution to pelvic morphology in the normal population has been poorly studied. Further studies, especially longitudinal studies, would be of great value to understand the variability of PI and its associated determinants.

## Discussion

Human beings are the only vertebrates to stand in an erect position with the trunk, hips, thighs, and legs simultaneously extended. Non-human primates can sometimes perform an occasional erect posture, but it is unstable and energy-consuming. Some species, such as the kangaroo, can walk with a stabilizing tail. Birds’ bipedalism is totally different from humans. The spinopelvic complex plays an essential role in the acquisition of upright posture.

Beyond this, there is a significant correlation between PI, lumbar spine curvature, and orientation of the acetabular cup [40]. Numerous studies indicated that a large PI may be associated with a great risk of lumbar spondylolisthesis, scoliosis, accelerated disc degeneration, disc herniation, and facet joint arthritis [41, 42]. Subjects with low PI are at higher risk of low back pain, discopathy, and sagittal imbalance. In recent years, hip surgeons have also paid great attention to PI. In patients with a low PI, there is more marked cross-sectional anteversion of the acetabulum. Theoretically, these patients have less capacity to adapt to sagittal imbalance and are more prone to femoroacetabular impingement syndrome at the femoral neck because overextension of the hips may occur easily in these patients. Inversely, in patients with a high PI value, the acetabular anteversion is less marked. Thus, these subjects have a better ability to compensate with a greater capacity for hip extension. However, they are at higher risk of anterior dislocation of the hip joint as well as hip osteoarthritis because of the anterior uncovering of the femoral heads. In these conditions, a large PI as well as a low PI can be a risk factor for spinal degenerative disease.

PI is generally accepted as a constant value, specific to each individual when bone maturity is reached. Different types of spinal profiles based on PI have their own degenerative mechanisms. Physicians should adopt specific behavior interventions for specific individual. However, the PI value varies from 35° to 85° in the normal population, which makes it difficult to define a normal PI range. The role of the spinal parameters and PI has been outlined. The pelvis is thought to act as the anchor for the vertebral column and a regulator of sagittal balance. The analysis of pelvic morphology and PI is essential to understand the impact of spinal deformity and plan an appropriate treatment regimen. LL can be predicted for a given patient by considering the preoperative PI value (LL = PI ± 9°). Rose et al. [43] proposed a formula (PI + TK − LL < 45°) as a guide to achieve sagittal balance at the time of deformity correction. Although operative management should be tailored to the patient, a general realignment goal has been established: SVA < 50 mm, PT < 22°, and PI-LL < ±9°. A meticulous analysis and customized treatment plan for each patient based on PI are required in the setting of spinal deformity correction. Notably, Lee et al. [44] observed increased PI in all patients with surgically corrected adult sagittal deformities, following surgical correction of the fixed LL, possibly because of sacroiliac joint motion. The analysis in this study revealed the disparity in PI after surgery to be significantly greater in non-sacropelvic fixation. This should serve as a reminder that in some situations, even when realignment of the lumbar spine is achieved appropriately, the increased PI after surgery during the follow-up period may lead to a PI-LL mismatch.

Some question whether it is an oversimplification to predict postoperative LL based on the PI value only, as this neglect the influence of the head, upper body, and lower limbs. Additionally, PI varies so greatly even in the normal population. Thus, other sagittal parameters must be taken into consideration when reconstructing the postoperative spinal alignment. Furthermore, we suggest that surgeons should also pay attention to the genetic background of the spinopelvic profile for spinal disease prevention.

We are not able to propose a specific reason for the great variability in PI, and further studies need to address the underlying causes of this variability. We can hypothesize that the spinal profile, pelvic size, and relative position of femoral heads and sacral endplate within the pelvis, even the value of the femoral anteversion angle, are closely related to PI. Additionally, the genetic background may also play an important role in the pelvic morphology and formation of PI.

## Conclusion

Since the pelvic morphological traits acquired from the evolution of bipedal locomotion may be passed on genetically, both PI and pelvic morphology may be genetically predetermined. A great variability in PI may already exist even before birth. Acquisition of erect posture and development of bipedal locomotion during growth may further reshape the pelvis. Postural or locomotor differences among individuals may result in varying pelvic sizes and position, contributing to the great variability in PI. This great variability should also serve as a reminder that the sagittal profile and bipedal locomotion mode, as well as the genetic background of every individual, are unique and specific, and clinicians should avoid universally applying broad generalizations. PI is an important parameter, and there are many theories behind its variability; however, due to the lack of quality research in this topic, we still do not have clear mechanistic answers.

## Abbreviations

ASD: Adjacent segmental disease
CP: Cerebral palsy
LL: Lumbar lordosis
PI: Pelvic incidence
PJF: Proximal junctional failure
PT: Pelvic tilt
PTH: Pelvic thickness
SD: Scheuermann’s disease
SS: Sacral slope
STK: Scheuermann’s thoracic kyphosis
STLK: Scheuermann’s thoracolumbar kyphosis
SVA: Sagittal vertical axis
THA: Total hip arthroplasty
TK: Thoracic kyphosis
